# {2-[Bis(3-methyl-1*H*-indol-2-yl)meth­yl]phenolato-κ*O*}dimeth­yl(tetra­hydro­furan-κ*O*)aluminium(III)

**DOI:** 10.1107/S1600536809040598

**Published:** 2009-10-13

**Authors:** Audrey C. Eisenberg, Joseph M. Tanski, Yutan D. Y. L. Getzler

**Affiliations:** a Departments of Chemistry & Biochemistry, Kenyon College, Gambier, OH 43214-9623, USA; b Department of Chemistry, Vassar College, 124 Raymond Ave., Box 406, Poughkeepsie, NY 12604-0744, USA

## Abstract

The title compound, [Al(CH_3_)_2_(C_25_H_21_N_2_O)(C_4_H_8_O)], was isolated as a minor component from a reaction mixture of the parent indolyl ligand and trimethyl­aluminum in tetra­hydro­furan. The ligands adopt a distorted tetra­hedral geometry around aluminium. Obvious hydrogen-bonding interactions are not present.

## Related literature

For general background to (indol­yl)methanes, see, see: Mason (2003 ▶); Mason *et al.* (2003 ▶). For related structures, see: Ziemkowska *et al.* (2007 ▶); Haddad *et al.* (2009 ▶). For patterns in hydrogen bonding, see: Steiner (2002 ▶).
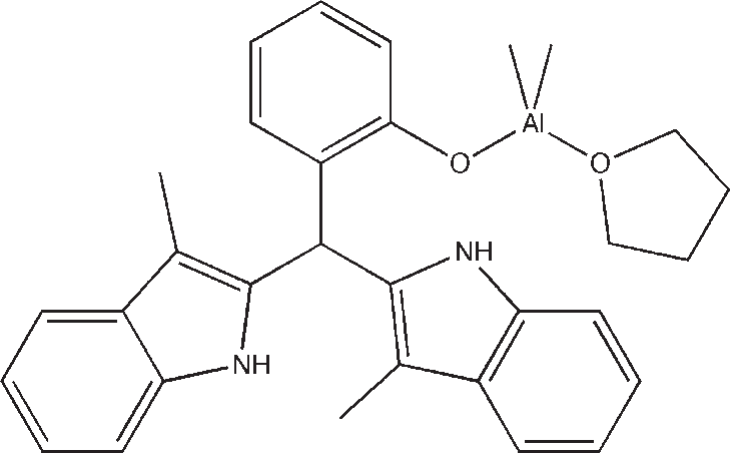


## Experimental

### 

#### Crystal data

[Al(CH_3_)_2_(C_25_H_21_N_2_O)(C_4_H_8_O)]
*M* *_r_* = 494.59
Monoclinic, 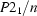

*a* = 11.2045 (1) Å
*b* = 19.4191 (3) Å
*c* = 12.5543 (2) Å
β = 98.537 (1)°
*V* = 2701.32 (6) Å^3^
*Z* = 4
Mo *K*α radiation
μ = 0.11 mm^−1^
*T* = 115 K
0.30 × 0.25 × 0.20 mm

#### Data collection

Bruker SMART CCD area-detector diffractometer
Absorption correction: multi-scan (*SADABS*; Bruker, 1999 ▶) *T*_min_ = 0.969, *T*_max_ = 0.979
55902 measured reflections
9864 independent reflections
7340 reflections with *I* > 2σ(*I*)
*R*_int_ = 0.039

#### Refinement

*R*[*F* ^2^ > 2σ(*F* ^2^)] = 0.051
*wR*(*F* ^2^) = 0.146
*S* = 1.04
9864 reflections
335 parameters
2 restraints
H atoms treated by a mixture of independent and constrained refinement
Δρ_max_ = 0.50 e Å^−3^
Δρ_min_ = −0.33 e Å^−3^

### 

Data collection: *SMART* (Bruker, 2001 ▶); cell refinement: *SAINT* (Bruker, 2001 ▶); data reduction: *SAINT*; program(s) used to solve structure: *SHELXS97* (Sheldrick, 2008 ▶); program(s) used to refine structure: *SHELXL97* (Sheldrick, 2008 ▶); molecular graphics: *Mercury* (Macrae *et al*., 2006 ▶); software used to prepare material for publication: *enCIFer* (Allen *et al.*, 2004 ▶).

## Supplementary Material

Crystal structure: contains datablocks I, global. DOI: 10.1107/S1600536809040598/pk2190sup1.cif

Structure factors: contains datablocks I. DOI: 10.1107/S1600536809040598/pk2190Isup2.hkl

Additional supplementary materials:  crystallographic information; 3D view; checkCIF report

## Figures and Tables

**Table:** 

Al—O1	1.7498 (9)
Al—O2	1.9097 (10)
Al—C9	1.9562 (16)
Al—C8	1.9588 (15)

**Table:** 

O1—Al—O2	99.27 (5)
O2—Al—C8	101.16 (6)
C9—Al—C8	121.34 (8)
C1—O1—Al	140.52 (8)

**Table 2:** Hydrogen-bond geometry (Å, °)

*D*—H⋯*A*	*D*—H	H⋯*A*	*D*⋯*A*	*D*—H⋯*A*
N2—H2⋯N1	0.869 (13)	2.703 (15)	3.2353 (14)	120.8 (12)
N1—H1⋯N2^i^	0.874 (13)	2.734 (14)	3.5391 (15)	153.8 (14)

Symmetry code: (i) 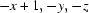.

**Fig. 1.**
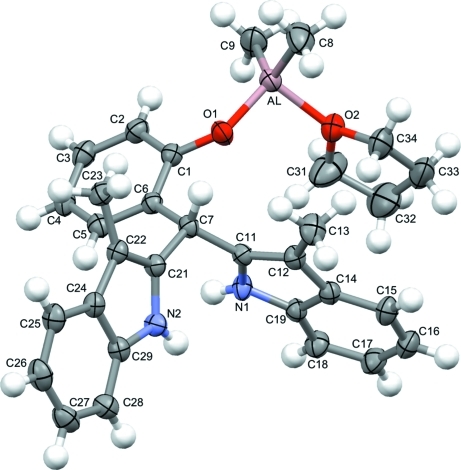
The molecular structure of (I), with atom labels and 50% probability displacement ellipsoids for non-H atoms.

**Fig. 2.**
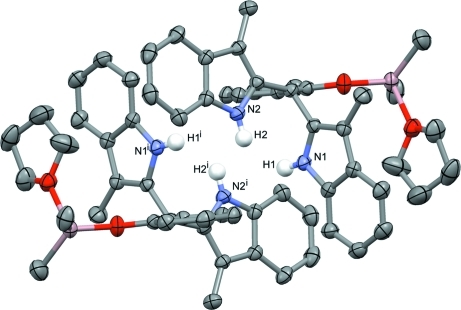
The molecular structure of (I), showing the possible weak intra- and intermolecular hydrogen bonds. Only the atoms involved are labeled and non-involved H atoms are omitted for clarity. Atoms with the 'i' superscript are related by the inversion operation (1-x,-y,-z).

## supplementary crystallographic information

### Comment

The molecule of the title complex (Fig. 1), has a distorted tetrahedral coordination geometry around aluminum with *X*—Al—*X* (*X* = C, O) angles ranging from 99.26 (5)° to 121.34 (8)°. The phenoxy(bisindolyl)methane ligand is coordinated to aluminum through a deprotonated phenol oxygen that is bent well out of the ideal *sp^3^* to 140.50 (8)°, consistent with significant π-donation to Al(III) from the phenoxide O. In the crystal structure (Fig. 2), weak intramolecular N—H···N interactions may stabilize the ligand conformations while weak intermolecular N—H···N interactions may stabilize crystal packing. The distances of these contacts [2.703 (15) Å and 2.734 (14) Å, respectively] are quite long (Steiner, 2002). However, they are slightly less then the sum of the relevant Van der Waals radii.

### Experimental

The ligand was synthesized by mixing two equivalents of 3-methylindole with one equivalent of salicylaldehyde in EtOH in the presence of an acid catalyst. The product was isolated by vacuum filtration (66.6%; mp 230–240 °C (dec.)); ^1^H NMR (300 MHz, DMSO-d6), 2.01 (6*H*, s), 6.22 (1*H*, s), 6.76 (1*H*, dt, J=1.2 H, 8.5 Hz), 6.85 (1*H*, d, J=5.4 Hz), 6.91–7.03 (4*H*, m), 7.08 (2*H*, d, J=7.4 Hz), 7.30 (2*H*, d, J=7.2 H), 7.4 (2*H*, d, J=6.9 H), 9.58 (1*H*, s), 10.31 (2*H*, s); ^13^C NMR (75 MHz, DMSO-d6) 8.34, 34.79, 105.93, 111.07, 115.07, 117.57, 18.09, 119.06, 120.26, 127.22, 127.74, 128.85, 129.57, 134.72, 135.36, 154.69.

Using standard Schlenk techniques, the free ligand was dissolved in THF and AlMe_3_ (2.0 *M* in heptane, 1 equivalent) was added dropwise *via* syringe. Upon reaction completion, solvent was removed under high vacuum and the residue was dissolved in a minimum of hot toluene. Upon sitting, a few small, colorless crystals appeared.

### Refinement

Hydrogen atoms on carbon were added geometrically and refined using a riding model, whereas hydrogen atoms on nitrogen were located in the difference map and refined semi-freely with the help of a distance restraint. *U*_iso_ values for hydrogen atoms were assigned to be 1.20 times the *U*_eq_ value of the atom to which they are attached, except for hydrogen atoms on methyl carbon atoms, which were assigned a *U*_iso_ of 1.50 times the *U*_eq_ of the methyl carbon atom to which they are attached.

### Crystal data

**Table:**

[Al(CH_3_)_2_(C_25_H_21_N_2_O)(C_4_H_8_O)]	*F*(000) = 1056
*M**_r_* = 494.59	*D*_x_ = 1.216 Mg m^−^^3^
Monoclinic, *P*2_1_/*n*	Mo *K*α radiation, λ = 0.71073 Å
Hall symbol: -P 2yn	Cell parameters from 6863 reflections
*a* = 11.2045 (1) Å	θ = 2.6–31.6°
*b* = 19.4191 (3) Å	µ = 0.11 mm^−^^1^
*c* = 12.5543 (2) Å	*T* = 115 K
β = 98.537 (1)°	Block, colorless
*V* = 2701.32 (6) Å^3^	0.30 × 0.25 × 0.20 mm
*Z* = 4	

### Data collection

**Table:**

Bruker SMART CCD area-detector diffractometer	9864 independent reflections
Radiation source: fine-focus sealed tube	7340 reflections with *I* > 2σ(*I*)
graphite	*R*_int_ = 0.039
φ and ω scans	θ_max_ = 32.7°, θ_min_ = 2.0°
Absorption correction: multi-scan (*SADABS*; Bruker, 1999)	*h* = −16→16
*T*_min_ = 0.969, *T*_max_ = 0.979	*k* = −29→29
55902 measured reflections	*l* = −18→19

### Refinement

**Table:**

Refinement on *F*^2^	Primary atom site location: structure-invariant direct methods
Least-squares matrix: full	Secondary atom site location: difference Fourier map
*R*[*F*^2^ > 2σ(*F*^2^)] = 0.051	Hydrogen site location: inferred from neighbouring sites
*wR*(*F*^2^) = 0.146	H atoms treated by a mixture of independent and constrained refinement
*S* = 1.04	*w* = 1/[σ^2^(*F*_o_^2^) + (0.0719*P*)^2^ + 0.8449*P*] where *P* = (*F*_o_^2^ + 2*F*_c_^2^)/3
9864 reflections	(Δ/σ)_max_ = 0.003
335 parameters	Δρ_max_ = 0.50 e Å^−^^3^
2 restraints	Δρ_min_ = −0.33 e Å^−^^3^

### Special details

**Table:**

Geometry. All e.s.d.'s (except the e.s.d. in the dihedral angle between two l.s. planes) are estimated using the full covariance matrix. The cell e.s.d.'s are taken into account individually in the estimation of e.s.d.'s in distances, angles and torsion angles; correlations between e.s.d.'s in cell parameters are only used when they are defined by crystal symmetry. An approximate (isotropic) treatment of cell e.s.d.'s is used for estimating e.s.d.'s involving l.s. planes.
Refinement. Refinement of *F*^2^ against ALL reflections. The weighted *R*-factor *wR* and goodness of fit *S* are based on *F*^2^, conventional *R*-factors *R* are based on *F*, with *F* set to zero for negative *F*^2^. The threshold expression of *F*^2^ > 2σ(*F*^2^) is used only for calculating *R*-factors(gt) *etc*. and is not relevant to the choice of reflections for refinement. *R*-factors based on *F*^2^ are statistically about twice as large as those based on *F*, and *R*- factors based on ALL data will be even larger.An extinction parameter (EXTI in SHELXL-97) refined to zero and was removed from the refinement.

### Fractional atomic coordinates and isotropic or equivalent isotropic displacement parameters (Å^2^)

**Table:**

	*x*	*y*	*z*	*U*_iso_*/*U*_eq_	
Al	1.01187 (3)	0.14505 (2)	0.20418 (3)	0.02200 (9)	
N1	0.57741 (10)	0.09740 (5)	0.12462 (8)	0.02006 (19)	
H1	0.5904 (14)	0.0533 (7)	0.1342 (13)	0.024*	
N2	0.47850 (9)	0.08185 (5)	−0.12975 (8)	0.01998 (19)	
H2	0.4441 (13)	0.0848 (8)	−0.0723 (11)	0.024*	
O1	0.90038 (8)	0.10626 (5)	0.11103 (7)	0.02398 (18)	
C1	0.86433 (10)	0.04405 (6)	0.07380 (9)	0.0192 (2)	
C2	0.93110 (11)	−0.01616 (6)	0.10154 (10)	0.0228 (2)	
H2B	1.0040	−0.0137	0.1509	0.027*	
C3	0.89153 (12)	−0.07922 (7)	0.05754 (10)	0.0250 (2)	
H3A	0.9377	−0.1196	0.0769	0.030*	
C4	0.78513 (12)	−0.08382 (6)	−0.01458 (10)	0.0247 (2)	
H4A	0.7578	−0.1271	−0.0441	0.030*	
C5	0.71913 (11)	−0.02421 (6)	−0.04303 (9)	0.0212 (2)	
H5A	0.6468	−0.0271	−0.0931	0.025*	
C6	0.75665 (10)	0.03961 (6)	0.00020 (9)	0.0178 (2)	
C7	0.68922 (10)	0.10618 (6)	−0.03436 (9)	0.0177 (2)	
H7A	0.7507	0.1398	−0.0526	0.021*	
C8	1.11591 (13)	0.08255 (8)	0.29899 (12)	0.0338 (3)	
H8A	1.0665	0.0467	0.3258	0.051*	
H8B	1.1586	0.1085	0.3599	0.051*	
H8C	1.1747	0.0612	0.2587	0.051*	
C9	1.07672 (16)	0.22760 (8)	0.14422 (14)	0.0394 (4)	
H9A	1.0100	0.2562	0.1095	0.059*	
H9B	1.1281	0.2141	0.0909	0.059*	
H9C	1.1246	0.2538	0.2022	0.059*	
C11	0.63063 (10)	0.13818 (6)	0.05412 (9)	0.0171 (2)	
C12	0.61172 (11)	0.20616 (6)	0.07482 (9)	0.0191 (2)	
C13	0.65235 (14)	0.26706 (7)	0.01607 (11)	0.0289 (3)	
H13A	0.6934	0.2511	−0.0430	0.043*	
H13B	0.7081	0.2950	0.0660	0.043*	
H13C	0.5821	0.2949	−0.0131	0.043*	
C14	0.54458 (10)	0.20819 (6)	0.16404 (9)	0.0190 (2)	
C15	0.50018 (12)	0.26175 (7)	0.22256 (10)	0.0254 (2)	
H15A	0.5110	0.3085	0.2039	0.030*	
C16	0.44039 (12)	0.24503 (7)	0.30784 (11)	0.0280 (3)	
H16A	0.4102	0.2809	0.3479	0.034*	
C17	0.42337 (12)	0.17622 (7)	0.33649 (11)	0.0271 (3)	
H17A	0.3821	0.1665	0.3956	0.033*	
C18	0.46554 (12)	0.12220 (7)	0.28017 (10)	0.0249 (2)	
H18A	0.4539	0.0756	0.2992	0.030*	
C19	0.52584 (11)	0.13945 (6)	0.19429 (9)	0.0192 (2)	
C21	0.59924 (10)	0.09616 (6)	−0.13497 (9)	0.0182 (2)	
C22	0.61934 (11)	0.09319 (6)	−0.23992 (9)	0.0188 (2)	
C23	0.73747 (11)	0.10156 (7)	−0.28045 (10)	0.0241 (2)	
H23A	0.8034	0.0993	−0.2198	0.036*	
H23B	0.7393	0.1463	−0.3164	0.036*	
H23C	0.7471	0.0646	−0.3317	0.036*	
C24	0.50504 (11)	0.07783 (6)	−0.30392 (9)	0.0189 (2)	
C25	0.46808 (12)	0.06826 (7)	−0.41469 (10)	0.0243 (2)	
H25A	0.5242	0.0723	−0.4641	0.029*	
C26	0.34840 (13)	0.05292 (7)	−0.45055 (10)	0.0287 (3)	
H26A	0.3228	0.0459	−0.5253	0.034*	
C27	0.26399 (12)	0.04754 (7)	−0.37887 (11)	0.0282 (3)	
H27A	0.1822	0.0374	−0.4062	0.034*	
C28	0.29754 (11)	0.05678 (7)	−0.26873 (10)	0.0239 (2)	
H28A	0.2405	0.0533	−0.2200	0.029*	
C29	0.41869 (11)	0.07144 (6)	−0.23296 (9)	0.0194 (2)	
O2	0.91231 (9)	0.18169 (5)	0.30148 (8)	0.0269 (2)	
C31	0.83901 (18)	0.13759 (9)	0.35943 (18)	0.0520 (5)	
H31A	0.8909	0.1064	0.4086	0.062*	
H31B	0.7834	0.1094	0.3084	0.062*	
C32	0.76950 (18)	0.18470 (12)	0.42187 (15)	0.0518 (5)	
H32A	0.7866	0.1739	0.4997	0.062*	
H32B	0.6818	0.1795	0.3977	0.062*	
C33	0.80926 (15)	0.25690 (10)	0.40136 (14)	0.0440 (4)	
H33A	0.7398	0.2889	0.3925	0.053*	
H33B	0.8701	0.2733	0.4613	0.053*	
C34	0.86274 (15)	0.25129 (8)	0.29894 (13)	0.0359 (3)	
H34A	0.8001	0.2577	0.2353	0.043*	
H34B	0.9270	0.2861	0.2969	0.043*	

### Atomic displacement parameters (Å^2^)

**Table:**

	*U*^11^	*U*^22^	*U*^33^	*U*^12^	*U*^13^	*U*^23^
Al	0.02055 (18)	0.02033 (18)	0.02432 (19)	−0.00075 (13)	0.00067 (14)	−0.00421 (14)
N1	0.0270 (5)	0.0147 (4)	0.0193 (4)	0.0000 (4)	0.0063 (4)	−0.0003 (3)
N2	0.0208 (5)	0.0241 (5)	0.0153 (4)	−0.0012 (4)	0.0038 (3)	−0.0005 (4)
O1	0.0225 (4)	0.0200 (4)	0.0274 (4)	0.0005 (3)	−0.0033 (3)	−0.0048 (3)
C1	0.0203 (5)	0.0190 (5)	0.0188 (5)	0.0007 (4)	0.0044 (4)	−0.0010 (4)
C2	0.0215 (5)	0.0229 (6)	0.0239 (5)	0.0033 (4)	0.0034 (4)	0.0003 (4)
C3	0.0291 (6)	0.0202 (5)	0.0268 (6)	0.0054 (5)	0.0079 (5)	0.0013 (4)
C4	0.0326 (6)	0.0180 (5)	0.0247 (6)	−0.0013 (5)	0.0086 (5)	−0.0032 (4)
C5	0.0238 (5)	0.0206 (5)	0.0192 (5)	−0.0028 (4)	0.0039 (4)	−0.0014 (4)
C6	0.0190 (5)	0.0188 (5)	0.0159 (5)	−0.0002 (4)	0.0042 (4)	−0.0004 (4)
C7	0.0194 (5)	0.0185 (5)	0.0149 (4)	−0.0009 (4)	0.0021 (4)	−0.0007 (4)
C8	0.0308 (7)	0.0337 (7)	0.0334 (7)	0.0073 (6)	−0.0068 (5)	−0.0077 (6)
C9	0.0462 (9)	0.0279 (7)	0.0473 (9)	−0.0103 (6)	0.0172 (7)	−0.0066 (6)
C11	0.0184 (5)	0.0177 (5)	0.0148 (4)	−0.0006 (4)	0.0011 (4)	0.0001 (4)
C12	0.0215 (5)	0.0170 (5)	0.0183 (5)	0.0006 (4)	0.0014 (4)	0.0009 (4)
C13	0.0397 (7)	0.0203 (6)	0.0283 (6)	−0.0012 (5)	0.0104 (5)	0.0036 (5)
C14	0.0200 (5)	0.0180 (5)	0.0184 (5)	0.0018 (4)	0.0009 (4)	−0.0004 (4)
C15	0.0299 (6)	0.0197 (6)	0.0268 (6)	0.0055 (5)	0.0052 (5)	−0.0012 (4)
C16	0.0296 (6)	0.0270 (6)	0.0288 (6)	0.0063 (5)	0.0085 (5)	−0.0047 (5)
C17	0.0281 (6)	0.0302 (7)	0.0250 (6)	0.0012 (5)	0.0100 (5)	−0.0019 (5)
C18	0.0294 (6)	0.0232 (6)	0.0236 (6)	−0.0012 (5)	0.0087 (5)	0.0000 (4)
C19	0.0205 (5)	0.0194 (5)	0.0176 (5)	0.0001 (4)	0.0024 (4)	−0.0021 (4)
C21	0.0193 (5)	0.0182 (5)	0.0169 (5)	0.0000 (4)	0.0026 (4)	0.0003 (4)
C22	0.0218 (5)	0.0186 (5)	0.0165 (5)	0.0016 (4)	0.0039 (4)	0.0011 (4)
C23	0.0241 (6)	0.0278 (6)	0.0217 (5)	0.0013 (5)	0.0073 (4)	0.0002 (4)
C24	0.0231 (5)	0.0169 (5)	0.0164 (5)	0.0019 (4)	0.0021 (4)	0.0007 (4)
C25	0.0316 (6)	0.0238 (6)	0.0169 (5)	0.0033 (5)	0.0019 (4)	0.0003 (4)
C26	0.0370 (7)	0.0274 (6)	0.0192 (5)	0.0014 (5)	−0.0044 (5)	−0.0009 (5)
C27	0.0267 (6)	0.0261 (6)	0.0288 (6)	−0.0021 (5)	−0.0053 (5)	0.0004 (5)
C28	0.0230 (6)	0.0229 (6)	0.0251 (6)	−0.0019 (4)	0.0011 (4)	0.0008 (4)
C29	0.0232 (5)	0.0171 (5)	0.0172 (5)	0.0004 (4)	0.0009 (4)	0.0003 (4)
O2	0.0309 (5)	0.0226 (4)	0.0278 (5)	0.0018 (4)	0.0059 (4)	−0.0036 (3)
C31	0.0533 (11)	0.0370 (9)	0.0742 (13)	0.0016 (8)	0.0375 (10)	0.0087 (8)
C32	0.0499 (10)	0.0722 (13)	0.0371 (9)	−0.0054 (9)	0.0191 (8)	−0.0110 (9)
C33	0.0340 (8)	0.0532 (10)	0.0432 (9)	0.0115 (7)	0.0006 (7)	−0.0234 (8)
C34	0.0371 (8)	0.0257 (7)	0.0451 (8)	0.0077 (6)	0.0065 (6)	−0.0065 (6)

### Geometric parameters (Å, °)

**Table:**

Al—O1	1.7498 (9)	C14—C19	1.4120 (16)
Al—O2	1.9097 (10)	C15—C16	1.3833 (19)
Al—C9	1.9562 (16)	C15—H15A	0.9500
Al—C8	1.9588 (15)	C16—C17	1.404 (2)
N1—C19	1.3841 (15)	C16—H16A	0.9500
N1—C11	1.3866 (15)	C17—C18	1.3866 (18)
N1—H1	0.874 (13)	C17—H17A	0.9500
N2—C29	1.3824 (15)	C18—C19	1.3955 (16)
N2—C21	1.3922 (15)	C18—H18A	0.9500
N2—H2	0.869 (13)	C21—C22	1.3705 (15)
O1—C1	1.3361 (14)	C22—C24	1.4384 (16)
C1—C2	1.4038 (17)	C22—C23	1.4958 (16)
C1—C6	1.4092 (16)	C23—H23A	0.9800
C2—C3	1.3887 (18)	C23—H23B	0.9800
C2—H2B	0.9500	C23—H23C	0.9800
C3—C4	1.3888 (19)	C24—C25	1.4032 (16)
C3—H3A	0.9500	C24—C29	1.4146 (16)
C4—C5	1.3914 (18)	C25—C26	1.3822 (19)
C4—H4A	0.9500	C25—H25A	0.9500
C5—C6	1.3928 (16)	C26—C27	1.403 (2)
C5—H5A	0.9500	C26—H26A	0.9500
C6—C7	1.5273 (16)	C27—C28	1.3896 (18)
C7—C11	1.5058 (15)	C27—H27A	0.9500
C7—C21	1.5072 (16)	C28—C29	1.3944 (17)
C7—H7A	1.0000	C28—H28A	0.9500
C8—H8A	0.9800	O2—C31	1.4541 (19)
C8—H8B	0.9800	O2—C34	1.4598 (17)
C8—H8C	0.9800	C31—C32	1.496 (3)
C9—H9A	0.9800	C31—H31A	0.9900
C9—H9B	0.9800	C31—H31B	0.9900
C9—H9C	0.9800	C32—C33	1.505 (3)
C11—C12	1.3680 (16)	C32—H32A	0.9900
C12—C14	1.4396 (16)	C32—H32B	0.9900
C12—C13	1.4999 (17)	C33—C34	1.501 (2)
C13—H13A	0.9800	C33—H33A	0.9900
C13—H13B	0.9800	C33—H33B	0.9900
C13—H13C	0.9800	C34—H34A	0.9900
C14—C15	1.4063 (17)	C34—H34B	0.9900
			
O1—Al—O2	99.27 (5)	C15—C16—H16A	119.3
O1—Al—C9	111.39 (7)	C17—C16—H16A	119.3
O2—Al—C9	103.09 (6)	C18—C17—C16	121.32 (12)
O1—Al—C8	116.16 (6)	C18—C17—H17A	119.3
O2—Al—C8	101.16 (6)	C16—C17—H17A	119.3
C9—Al—C8	121.34 (8)	C17—C18—C19	116.94 (12)
C19—N1—C11	109.02 (10)	C17—C18—H18A	121.5
C19—N1—H1	124.6 (10)	C19—C18—H18A	121.5
C11—N1—H1	124.7 (10)	N1—C19—C18	129.94 (11)
C29—N2—C21	108.92 (9)	N1—C19—C14	107.16 (10)
C29—N2—H2	125.1 (10)	C18—C19—C14	122.90 (11)
C21—N2—H2	125.4 (10)	C22—C21—N2	109.66 (10)
C1—O1—Al	140.52 (8)	C22—C21—C7	128.77 (11)
O1—C1—C2	122.96 (11)	N2—C21—C7	121.34 (10)
O1—C1—C6	118.01 (10)	C21—C22—C24	106.62 (10)
C2—C1—C6	118.99 (11)	C21—C22—C23	127.05 (11)
C3—C2—C1	120.59 (12)	C24—C22—C23	126.30 (10)
C3—C2—H2B	119.7	C22—C23—H23A	109.5
C1—C2—H2B	119.7	C22—C23—H23B	109.5
C2—C3—C4	120.57 (12)	H23A—C23—H23B	109.5
C2—C3—H3A	119.7	C22—C23—H23C	109.5
C4—C3—H3A	119.7	H23A—C23—H23C	109.5
C3—C4—C5	119.07 (12)	H23B—C23—H23C	109.5
C3—C4—H4A	120.5	C25—C24—C29	118.93 (11)
C5—C4—H4A	120.5	C25—C24—C22	133.50 (11)
C4—C5—C6	121.48 (11)	C29—C24—C22	107.57 (10)
C4—C5—H5A	119.3	C26—C25—C24	118.73 (12)
C6—C5—H5A	119.3	C26—C25—H25A	120.6
C5—C6—C1	119.30 (11)	C24—C25—H25A	120.6
C5—C6—C7	122.31 (10)	C25—C26—C27	121.40 (12)
C1—C6—C7	118.28 (10)	C25—C26—H26A	119.3
C11—C7—C21	111.15 (9)	C27—C26—H26A	119.3
C11—C7—C6	113.02 (9)	C28—C27—C26	121.34 (12)
C21—C7—C6	111.72 (9)	C28—C27—H27A	119.3
C11—C7—H7A	106.8	C26—C27—H27A	119.3
C21—C7—H7A	106.8	C27—C28—C29	116.96 (12)
C6—C7—H7A	106.8	C27—C28—H28A	121.5
Al—C8—H8A	109.5	C29—C28—H28A	121.5
Al—C8—H8B	109.5	N2—C29—C28	130.16 (11)
H8A—C8—H8B	109.5	N2—C29—C24	107.20 (10)
Al—C8—H8C	109.5	C28—C29—C24	122.64 (11)
H8A—C8—H8C	109.5	C31—O2—C34	108.35 (11)
H8B—C8—H8C	109.5	C31—O2—Al	121.84 (9)
Al—C9—H9A	109.5	C34—O2—Al	125.98 (9)
Al—C9—H9B	109.5	O2—C31—C32	106.18 (15)
H9A—C9—H9B	109.5	O2—C31—H31A	110.5
Al—C9—H9C	109.5	C32—C31—H31A	110.5
H9A—C9—H9C	109.5	O2—C31—H31B	110.5
H9B—C9—H9C	109.5	C32—C31—H31B	110.5
C12—C11—N1	109.66 (10)	H31A—C31—H31B	108.7
C12—C11—C7	129.43 (10)	C31—C32—C33	106.83 (14)
N1—C11—C7	120.74 (10)	C31—C32—H32A	110.4
C11—C12—C14	106.73 (10)	C33—C32—H32A	110.4
C11—C12—C13	126.92 (11)	C31—C32—H32B	110.4
C14—C12—C13	126.36 (11)	C33—C32—H32B	110.4
C12—C13—H13A	109.5	H32A—C32—H32B	108.6
C12—C13—H13B	109.5	C34—C33—C32	104.18 (13)
H13A—C13—H13B	109.5	C34—C33—H33A	110.9
C12—C13—H13C	109.5	C32—C33—H33A	110.9
H13A—C13—H13C	109.5	C34—C33—H33B	110.9
H13B—C13—H13C	109.5	C32—C33—H33B	110.9
C15—C14—C19	118.71 (11)	H33A—C33—H33B	108.9
C15—C14—C12	133.87 (11)	O2—C34—C33	104.33 (13)
C19—C14—C12	107.40 (10)	O2—C34—H34A	110.9
C16—C15—C14	118.70 (12)	C33—C34—H34A	110.9
C16—C15—H15A	120.6	O2—C34—H34B	110.9
C14—C15—H15A	120.6	C33—C34—H34B	110.9
C15—C16—C17	121.43 (12)	H34A—C34—H34B	108.9
			
O2—Al—O1—C1	−114.62 (13)	C12—C14—C19—N1	−1.19 (13)
C9—Al—O1—C1	137.30 (13)	C15—C14—C19—C18	−0.29 (18)
C8—Al—O1—C1	−7.24 (15)	C12—C14—C19—C18	178.64 (11)
Al—O1—C1—C2	−7.9 (2)	C29—N2—C21—C22	−1.74 (14)
Al—O1—C1—C6	174.60 (10)	C29—N2—C21—C7	−176.69 (10)
O1—C1—C2—C3	−177.73 (11)	C11—C7—C21—C22	153.80 (12)
C6—C1—C2—C3	−0.25 (18)	C6—C7—C21—C22	−78.94 (15)
C1—C2—C3—C4	−0.09 (19)	C11—C7—C21—N2	−32.31 (15)
C2—C3—C4—C5	0.61 (19)	C6—C7—C21—N2	94.95 (13)
C3—C4—C5—C6	−0.81 (18)	N2—C21—C22—C24	1.35 (13)
C4—C5—C6—C1	0.48 (17)	C7—C21—C22—C24	175.81 (11)
C4—C5—C6—C7	176.69 (11)	N2—C21—C22—C23	−176.55 (11)
O1—C1—C6—C5	177.66 (10)	C7—C21—C22—C23	−2.1 (2)
C2—C1—C6—C5	0.05 (16)	C21—C22—C24—C25	−179.78 (13)
O1—C1—C6—C7	1.30 (15)	C23—C22—C24—C25	−1.9 (2)
C2—C1—C6—C7	−176.31 (10)	C21—C22—C24—C29	−0.48 (13)
C5—C6—C7—C11	113.18 (12)	C23—C22—C24—C29	177.43 (11)
C1—C6—C7—C11	−70.57 (13)	C29—C24—C25—C26	0.00 (18)
C5—C6—C7—C21	−13.06 (15)	C22—C24—C25—C26	179.24 (13)
C1—C6—C7—C21	163.18 (10)	C24—C25—C26—C27	0.7 (2)
C19—N1—C11—C12	−1.64 (13)	C25—C26—C27—C28	−0.6 (2)
C19—N1—C11—C7	−177.26 (10)	C26—C27—C28—C29	−0.1 (2)
C21—C7—C11—C12	−86.14 (15)	C21—N2—C29—C28	−179.01 (12)
C6—C7—C11—C12	147.32 (12)	C21—N2—C29—C24	1.39 (13)
C21—C7—C11—N1	88.52 (12)	C27—C28—C29—N2	−178.71 (12)
C6—C7—C11—N1	−38.02 (14)	C27—C28—C29—C24	0.83 (18)
N1—C11—C12—C14	0.85 (13)	C25—C24—C29—N2	178.86 (11)
C7—C11—C12—C14	175.98 (11)	C22—C24—C29—N2	−0.56 (13)
N1—C11—C12—C13	−178.94 (12)	C25—C24—C29—C28	−0.78 (18)
C7—C11—C12—C13	−3.8 (2)	C22—C24—C29—C28	179.80 (11)
C11—C12—C14—C15	178.93 (13)	O1—Al—O2—C31	61.17 (14)
C13—C12—C14—C15	−1.3 (2)	C9—Al—O2—C31	175.83 (14)
C11—C12—C14—C19	0.22 (13)	C8—Al—O2—C31	−58.00 (14)
C13—C12—C14—C19	−179.99 (12)	O1—Al—O2—C34	−94.15 (11)
C19—C14—C15—C16	0.32 (18)	C9—Al—O2—C34	20.51 (13)
C12—C14—C15—C16	−178.27 (13)	C8—Al—O2—C34	146.67 (11)
C14—C15—C16—C17	−0.1 (2)	C34—O2—C31—C32	−17.7 (2)
C15—C16—C17—C18	−0.2 (2)	Al—O2—C31—C32	−176.89 (12)
C16—C17—C18—C19	0.2 (2)	O2—C31—C32—C33	−2.3 (2)
C11—N1—C19—C18	−178.09 (12)	C31—C32—C33—C34	20.5 (2)
C11—N1—C19—C14	1.73 (13)	C31—O2—C34—C33	30.70 (17)
C17—C18—C19—N1	179.81 (12)	Al—O2—C34—C33	−171.24 (9)
C17—C18—C19—C14	0.01 (19)	C32—C33—C34—O2	−30.95 (17)
C15—C14—C19—N1	179.87 (11)		

### Hydrogen-bond geometry (Å, °)

**Table:**

*D*—H···*A*	*D*—H	H···*A*	*D*···*A*	*D*—H···*A*
N2—H2···N1	0.87 (1)	2.70 (2)	3.2353 (14)	121 (1)
N1—H1···N2^i^	0.87 (1)	2.73 (1)	3.5391 (15)	154 (1)

Symmetry codes: (i) −*x*+1, −*y*, −*z*.
